# Gradients of salinity and plant community richness and diversity in two different Mediterranean coastal ecosystems in NW Sardinia

**DOI:** 10.3897/BDJ.9.e71247

**Published:** 2021-11-19

**Authors:** Alfredo Maccioni, Luisa Canopoli, Valeria Cubeddu, Elisabetta Cucca, Simone Dessena, Samuele Morittu, Rossella Filigheddu, Bachisio Mario Padedda, Emmanuele Farris

**Affiliations:** 1 Department of Chemistry and Pharmacy, University of Sassari, Via Piandanna 4, - 07100, Sassari, Italy Department of Chemistry and Pharmacy, University of Sassari, Via Piandanna 4, - 07100 Sassari Italy; 2 School of Water, Energy and Environment, Cranfield University, Cranfield, Bedfordshire, United Kingdom School of Water, Energy and Environment, Cranfield University Cranfield, Bedfordshire United Kingdom; 3 Department of Architecture, Design and Urban Planning, University of Sassari, Piazza Duomo 6, I-07041, Alghero, Italy Department of Architecture, Design and Urban Planning, University of Sassari, Piazza Duomo 6, I-07041 Alghero Italy

**Keywords:** biodiversity, psammophilous vegetation and flora, rocky vegetation and flora, soil salinity, spatial distribution pattern, Sardinia

## Abstract

This study aimed to test if differences in soil salinity, plant richness and diversity were significantly affected by habitat, site and distance from the seashore at three sandy and three rocky coastal sites in north-western Sardinia.

Each site has been divided into three belts placed at an equal distance of 50 m from the shoreline. We measured soil salinity using a probe and vascular plants richness and diversity using linear transects at all sites. Average soil salinity varied from 0.115 g/l to 0.180 g/l; it was higher in the rocky habitats than in the sandy ones. A total of 21 species were found per transect/site at the rocky sites and 30 species per transect/site at the sandy sites, with an average of Shannon and Weaver's Diversity Index of 1.8 per each belt at each site. These data confirm that, also in the Mediterranean islands, there are coastal gradients of soil salinity from the seashore to inland areas and that also vascular plant richness and diversity are influenced by the distance from the sea. Soil salinity was strongly affected by the type of habitat, being average at the rocky coasts and negligible at the sandy shores. The site effect was not significant for both soil salinity and plant richness and diversity.

## Introduction

Studies on the effects of biotic and abiotic factors on biodiversity are a central topic in plant ecology (Klanderud et al. 2015). Assessing the main factors which affect the distribution and abundance of vascular plants in a habitat is of crucial importance for identifying the plant populations and plant communities’ structure and dynamics. The main abiotic factors that regulate the presence and distribution of vascular plants in coastal environments are climate (Thompson 2020), nutrients and soil chemistry (Lane et al. 2008, Gormally and Donovan 2010), moisture (Griffiths 2006, Gormally and Donovan 2010) and wind (Lortie and Cushman 2007, Gormally and Donovan 2010). Several studies have investigated the responses of vascular plant species to burial and salt spray in sandy environments (Donnelly and Pammenter 1983,Rozema et al. 1985, Maun and Perumal 1999, Wilson and Sykes 1999, Owen et al. 2004, Gormally and Donovan 2010), identifying them as the most important factors for determining environmental gradients on coastal dunes (Maun and Perumal 1999, Gormally and Donovan 2010) and controlling the zonation of psammophilous vegetation (Griffiths 2006, Lane et al. 2008, Fenu et al. 2013). The salt spray generally causes a reduction in the growth of the dune plants acting directly (Griffiths 2006) or in association with the burial process (Gormally and Donovan 2010) and wind exposure (Donnelly and Pammenter 1983). In addition, biotic interactions (Farris et al. 2017), disturbance (Blondel 2006) and historical and geographical factors (Thompson 2020) should be considered.

Abiotic drivers are recognised to play a major role in determining plant diversity in Mediterranean coastal dunes (Sperandii et al. 2019). Spatio-temporal gradients of soil salinity (i.e. the content of soluble salts in the soil, see Keren 2000) and water determine the zonation and distribution of plant taxa of coastal vegetation and habitats of salt marshes and endorheic basins (Llorens et al. 2018). Furthermore, topography determines the levels of salt concentration and, consequently, plant zoning in these areas (Bockelmann et al. 2002, Poeta et al. 2014), particularly in Mediterranean ecosystems (Acosta et al. 2003, Acosta et al. 2007).

Coastal cliffs and dunes can be considered as a set of different vegetation types which are arranged usually in parallel lines, according to a gradient of distance parallel to the coastline, from the sea to the inland: the zone close to the water edge is usually without vascular plants; a zone occupied host by annual halo-nitrophilous communities follows, then perennial grasslands on dunes or chasmophytic plants on cliffs complete the spatial succession. In a mosaic with the perennial vegetation, annual communities develop, characterised by the presence of small herbs with short life-cycles (Gratani et al. 2007). The continental (inland) part of dunes and cliffs usually hosts scrub communities characterised by the presence of several species of the genus *Juniperus* L. (in Sardinia, mainly *J.macrocarpa* Sm. and *J.turbinata* Guss., see Biondi et al. 2001, Farris et al. 2017). Conventionally, on dunes, three parts are recognised from the seashore to inland: embryo, mobile and fixed dune (Ruocco et al. 2014, Angiolini et al. 2018), also named as fore-dune, mid-dune and back-dune by other authors (Cusseddu et al. 2016), respectively.

It has been experimentally demonstrated that the different environmental factors that limit the growth of the psammophilous plants (soil moisture and nutrients, exposure to wind, burial, salt spray and salinity of the soil) change along the dunes following the distance from the sea (Wilson and Sykes 1999): in general, soil moisture and organic matter increase from coastal to the inland dune, while pH and salinity decrease (Angiolini et al. 2013). From the fore-dune to the back-dune, the vegetation is less exposed to extreme conditions and is always less tolerant to different stresses (Acosta et al. 2009, Fenu et al. 2013). Soil salinity is usually a neglected component, even when other soil factors are taken into consideration, except in the ecology of deserts and wetlands (Bui 2013). Regarding plant biodiversity on rocky coasts, the distribution of vegetation is influenced, not only by the scarcity of substrate and wind (limiting factors), but also by the high salinity in the soil (Jung et al. 2019), which is stronger near the shoreline (Pignatti 2012). Salinity in these soils has never been measured and, consequently, its role in shaping plant richness and diversity has been underestimated or just supposed (Jung et al. 2019). It was already highlighted that, in Mediterranean coastal cliff ecosystems, the stressful combination of high irradiance, high temperatures and low rainfall typical of the summer season may have been intensified by the shallow soils which display a poor water storage capacity (Ciccarelli et al. 2016).

Although Mediterranean coastal environments are considered a global biodiversity hotspot (Médail and Quézel 1999, Myers et al. 2000, Thompson 2020), few studies have been carried out measuring soil salinity in coastal dunes and cliffs and, therefore, a cognitive gap exists regarding the description of soil salinity gradients and the comprehension of the effects of spatial attributes (habitat, site and distance from the seashore) on these gradients and on plant diversity patterns in Mediterranean coastal environments.

In this research, therefore, we aimed to: 1) measure soil salinity in sandy and rocky Mediterranean insular coastal environments; 2) verify if also species richness and diversity of vascular plants showed spatial gradients similar to soil salinity; and 3) evaluate if the measured gradients (of soil salinity and plant richness and diversity) are significantly affected by the different habitat (rocky vs. sandy), by the site (three sites per habitat), by the distance from the seashore (three fixed distances) or by a combination of the three factors.

## Materials and Methods

### Study area and vegetation

**Figure 1.**
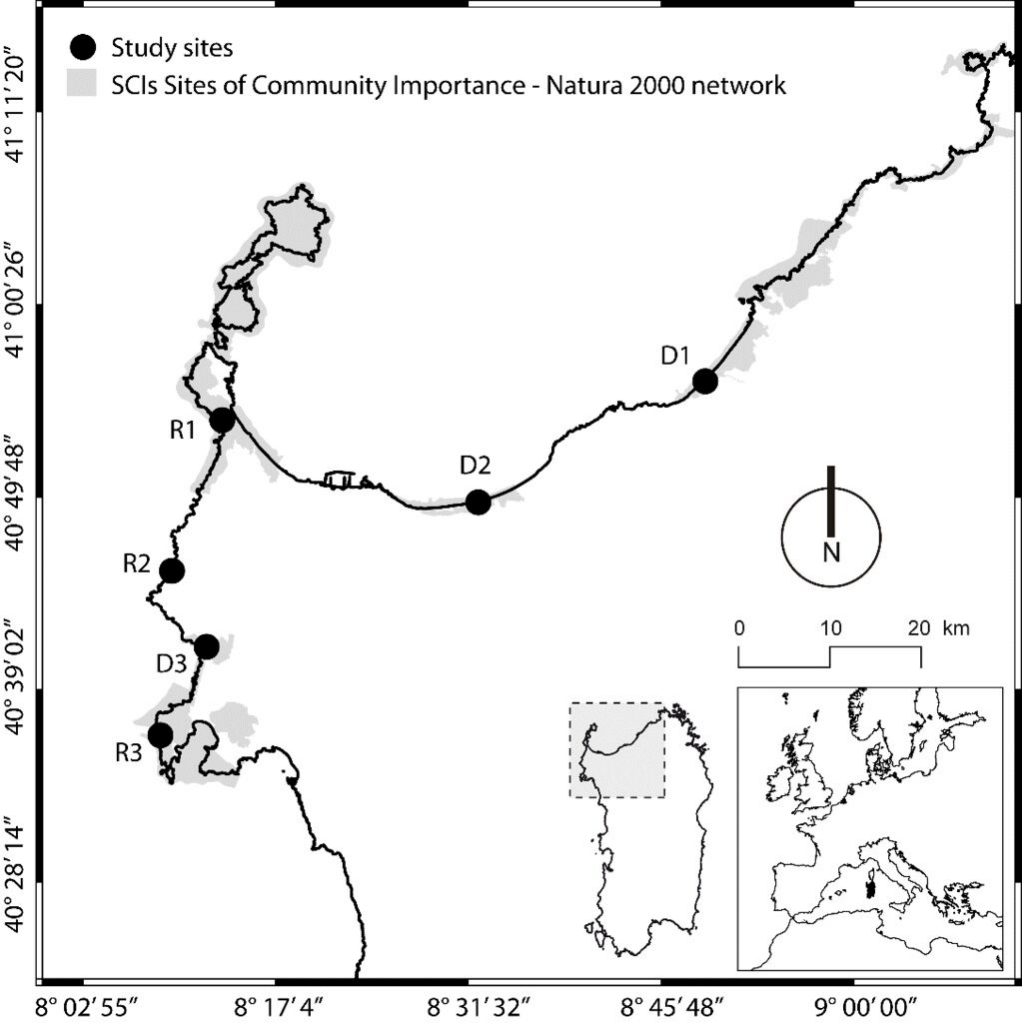
Location of the study area and sampling sites, north-western coast of Sardinia: R1 = Coscia di Donna; R2 = La Frana; R3 = Cala Barca; D1 = Li Junchi; D2 = Platamona; D3 = Porto Ferro; R = rocky sites; D = dune sites.

Mediterranean insular systems are considered hotspots of plant biodiversity of global importance (Mayer 1995,Médail and Quézel 1999, Myers et al. 2000). Sardinia, the second largest island of the Mediterranean Basin at 24,089 km and with 1,897 km of coastline, has an indigenous flora of ca. 2,500 vascular plants (see Farris et al. (2018) for a synthesis), of which 260 are Sardinian-Corsican endemics, mainly located in harsh habitats (Bacchetta et al. 2012). Marignani et al. (2014) consider that 8% of the Island’s surface has a high value for plant diversity conservation.The study area was located in north-western Sardinia (Fig. 1). For this study, we selected six coastal sites (Fig. 1), of which three are rocky (R) and three sandy (D), all located on the seashore and all having a typical Mediterranean climate with prolonged summer drought and mild winter: the average annual temperature is about 16°C and the average annual rainfall ranges from 500 to 600 mm. All study sites are included in the Mediterranean Pluviseasonal Oceanic bioclimate (Canu et al. 2015). At the study sites, plant communities belong to the typical Mediterranean coastal evergreen vegetation, described in previous papers (Molinier and Monier 1955, Mayer 1995, Biondi et al. 2001, Bacchetta et al. 2009)

### Sampling design

For this study, we selected three rocky (R) and three sandy (D) sites in north-western Sardinia (Italy, see Fig. 1): each study site was divided into three belts parallel to the coastline: the first 0 - 50 m from the shoreline (Belt 1), the second 51 - 100 m (Belt 2) and the third 101 - 150 m (Belt 3). For each site and at each Belt, soil salinity, vascular plant richness and diversity were measured.

We sampled on a gradual elevation gradient in the sandy sites (0-5 m a.s.l.), whereas in the rocky sites, having a steeper shoreline than dunes, we sampled on the summit plateau and not on the cliffs. Therefore, there were no significant differences in the elevation above the sea amongst the three Belts at each site. Furthermore, differences in average elevations above the sea amongst the rocky sites were negligible (10-30 m a.s.l.). We did not expect any tidal influence on soil salinity, because, in the Mediterranean Basin, average tidal height is about 0.2-0.3 m (Fenu et al. 2013).

### Soil salinity

Soil salinity was measured through the use of a probe manufactured by the company HANNA Instruments, model HI 993310. The probe operates in the conductivity range from 0.00 to 19.99 mS/cm and has a resolution of 0.01 mS/cm. The in situ measurements of the conductivity of the upper soil were carried out using the sensor probe following the manufacturer’s instructions, inserting it in the soil after wetting with 0.5 litres of distilled water and at a depth sufficient to ensure adequate soil moisture (up to 4 - 5 cm) and also because of the skeletal nature of the studied rocky soils.

The soil salinity data can be read a few seconds after the measurement on a small display connected with the probe and are given as g/l. To classify the obtained values as low, medium, high or very high, we followed the classification proposed by the U.S. Salinity Laboratory Staff (1954).

Soil salinity, expressed as g/l, was sampled at the three belts of each site during 2013 in the months of April (T1), August (T2) and December (T3). At each Belt, 10 measurements of soil salinity were taken, for a total of 30 measurements at each study site each time, so overall, we took 540 measurements for this study. All measurements per Belt were performed along the Belt section of 50 m in about an equal distance of no less than 4metres from each other.

### Species richness and community diversity

We determined species’ richness and community diversity by using the method of linear transects (theoretically, a frame quadrat of an infinitesimally small area, i.e. a point in linear transect), useful to study small size vegetation and especially in cases where it is difficult to distinguish individual plants (Bullock 1996), like the case of the vegetation under study.Within each Belt, at each study site, linear transects were carried out perpendicularly to the coastline: the vegetation was sampled each 50 cm along the transect, for a total 100 rod fall points. For each study site, five transects per Belt were executed; therefore, a total of 15 transects were performed at each study site and overall 90 transects (9,000 rod fall points) were carried out.

### Statistical analyses

Vegetation data of transects were used to calculate Shannon-Weaver Diversity Index (H'; Shannon and Weaver 1949).Three-ways ANOVAs were used in order to assess significant differences in the salinity of the substrates, Shannon and Weaver Index and number of species, between habitats (rocky vs. sandy), sites and distances from the sea (Belts): factor 1 was habitat (two levels: rocky vs. sandy) and was considered orthogonal and fixed; factor 2 was Site (three sites per each habitat) and was considered nested to Habitat and random; factor 3 was Belt (three belts at each site) and was considered nested in Habitat × Site. For soil salinity, we performed three different ANOVAs, one for each sampling time. Prior to analyses, the homogeneity of variances was tested by Cochran’s C-test and, whenever necessary, data were appropriately transformed. Post-hoc Student Newman - Keuls tests (SNK, p < 0.05) were run to compare the means of significant factors. ANOVAs were performed using the GMAV5 software package.

## Results

### Soil salinity

Table 1 shows the average soil salinity at the six study sites. Considering conventional ranges (U.S. Salinity Laboratory Staff 1954), we always found soil salinity to be medium at rocky sites and low (indeed very low) at sandy sites. As expected, we always found higher soil salinity at Belt 1 (closer to the sea) than in Belts 2 and 3.

**Figure 2.**
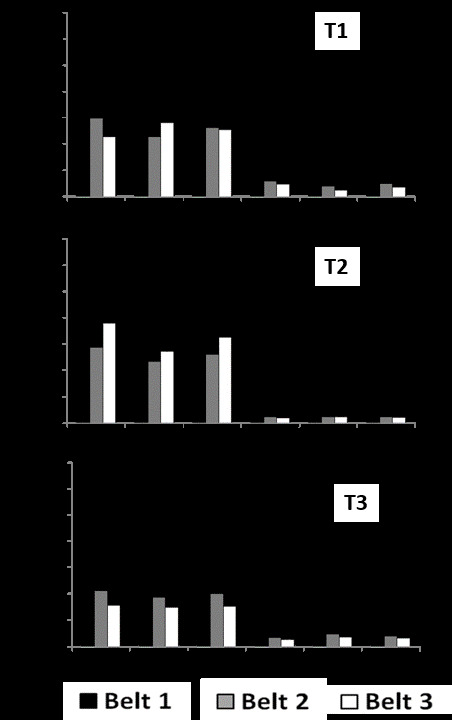
Soil salinity (g/l) at two habitats (R = rocky; D = sandy); three sites per habitat (R1 = Coscia di Donna; R2 = La Frana; R3 = Cala Barca; D1 = Li Junchi; D2 = Platamona; D3 = Porto Ferro) and three belts per site (Belt 1 = 0-50 m from the sea; Belt 2 = 51-100 m; Belt 3 = 101-150 m), sampled at three times in 2013 (T1 = April; T2 = August; T3 = December). A total of 10 measurements were taken at each combination Time × Habitat × Site × Belt. ^1^Classification of soil salinity according to U.S. Salinity Laboratory Staff 1954: low= < 0.2 [salt concentration (g/l)] and < 0.25 [electrical conductivity (dS/m)]; medium= 0.2-0.5 [salt concentration (g/l)] and 0.25-0.75 [electrical conductivity (dS/m)]; high=0.5-1.5 [salt concentration (g/l)] and 0.75-2.25 [electrical conductivity (dS/m)]; very high= 1.5-3.0 [salt concentration (g/l)] and 2.25-5.50 [electrical conductivity (dS/m)].

Extreme values ranged from 0.551±0.08 g/l at the Belt 1/site R1/time 2 (classified as “high” according to Table 1) and 0.011±0.005 g/l recorded at Belt 1/site D2/time 2 and 0.010±0.002 g/l at Belt 1/site D1/time 3 (Fig. 2).

ANOVAs emphasise a significant effect of the habitat and the distance from the sea on the soil salt concentration, whereas the site was not a significant factor at any time (Table 2).

At the time 1, the SNK test for the factor Habitat (SE = 0.0814) showed that dunes had a significantly lower soil salinity than rocky shores. SNK for the factor Belt (nested in Habitat x Site, SE = 0.1876) highlighted at all the three rocky sites that Belt 1 (0-50 m from the seashore) had a higher salinity than Belt 2 and Belt 3, which showed similar levels (Belt1>Belt2=Belt3), whereas, at the three sandy sites, no significant differences were found in soil salinity amongst the three belts (Belt1=Belt2=Belt3).

At the time 2, the SNK test for the factor Habitat (SE = 0.1549) presented a significantly lower soil salinity in dunes than rocky shores. SNK for the factor Belt (nested in Habitat x Site, SE = 0.2034) at the rocky site R1 showed a higher salinity at Belt 1 (0-50 m from the seashore) than Belt 2 and Belt 3, which showed similar levels (Belt1>Belt2=Belt3), whereas, at the other two rocky sites (R2 and R3) and at the three sandy sites, no significant differences were identified in soil salinity amongst the three belts (Belt1=Belt2=Belt3).

At the time 3, the SNK test for the factor Habitat (SE = 0.0666) showed that dunes had a significantly lower soil salinity than rocky shores. SNK for the factor Belt (nested in Habitat x Site, SE = 0.1462) highlighted at the rocky site R2 that Belt 1 (0-50 m from the seashore) had a higher salinity than Belt 2 and Belt 3, which showed similar levels (Belt1>Belt2=Belt3), whereas, at the other two rocky sites (R1 and R3) and at two sandy sites, no significant differences were found in soil salinity amongst the three Belts (Belt1=Belt2=Belt3). Interestingly, at site D1, Belt1 had a significantly lower soil salinity than the other two Belts (Belt1<Belt2=Belt3).

### Species richness and community diversity

We obtained a list of 89 vascular plants (floristic data are given in Suppl. material 1), considering all 90 point transects carried out in the studied coastal sites. We assessed a dominance of annual plants (Therophytes – T, 24%), followed by Chamaephytes (Ch, 21%), herbaceous perennial plants (Hemicryptophytes – H, 19%), Geophytes (G, 16%), nano-Phanaerophytes (NP, 11%) and Phanaerophytes (P, 9%). Dominant chorotypes were Steno-Mediterranean for 50%, Euro-Mediterranean for 17% and endemic for 11%.

**Figure 3.**
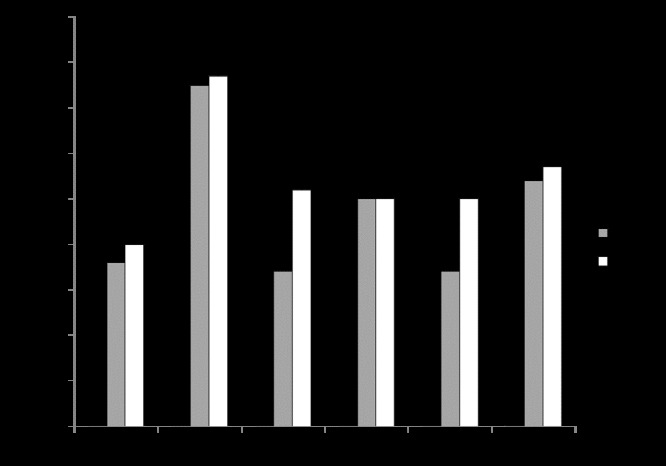
Number of vascular plant species at three rocky (R1 = Coscia di Donna, R2 = La Frana, R3 = Cala Barca) and three sandy (D1 = Li Junchi, D2 = Platamona, D3 = Porto Ferro) sites, censused at three belts (Belt 1 = 0-50 m from the sea; Belt 2 = 51-100 m; Belt 3 = 101-150 m), by means of five point transects (every 100 points) per belt.

In the rocky sites (R1, R2 and R3), we found 22, 18 and 23 species, respectively, while in the sandy sites (D1, D2 and D3), we found 33, 30 and 27 species, respectively. We found an average of 21 species per site at the rocky sites and 30 species per site at the sandy sites. The highest average number of species was found in Belt 3 with 10 species/transect (higher value was found in Belt 3/site R2 with 15 species/transect), followed by Belt 2 with 9 species/transect and Belt 1 with 7 species/transect (Fig. 3).

**Figure 4.**
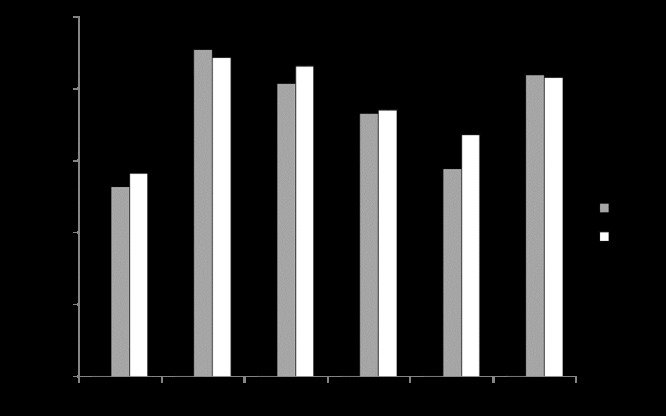
Shannon and Weaver Biodiversity Index (H’) of vascular plants at three rocky (R1 = Coscia di Donna, R2 = La Frana, R3 = Cala Barca) and three sandy (D1 = Li Junchi, D2 = Platamona, D3 = Porto Ferro) sites, censused at three Belts (Belt 1 = 0-50 m from the sea; Belt 2 = 51-100 m; Belt 3 = 101-150 m), by means of five point transects (every 100 points) per belt.

Shannon and Weaver's Diversity Index (Shannon and Weaver 1949) indicates an average H’ = 1.8 per Belt. Rocky sites had an average H’ per Belt higher than sandy sites (1.9 vs. 1.7). Noteworthy, the majority of sites showed an increasing H’ from Belt 1 to Belt 2 and Belt 3 (Fig. 4): H’ was on average 1.62 at Belt 1, 1.8 at Belt 2 and 1.9 at Belt 3.

ANOVA for the number of species (Table 3) showed the significant effect of the Belt. The SNK test for the factor Belt (nested in Habitat × Site, SE = 0.8192) did not highlight any significant differences in the number of species amongst Belts at the rocky sites R1 and R2, whereas at site R3, an increasing number of specieswas recorded from Belt 1 to Belt 3 (Belt1<Belt2<Belt3). Similarly, at the two sandy sites D1 and D2, no significant differences were highlighted in the number of species amongst the three Belts (Belt1=Belt2=Belt3), whereas at the sandy site D3, Belt 1 showed a significantly lower number of vascular plant species with respect to the other two Belts (Belt1<Belt2=Belt3).

ANOVA for the Shannon and Weaver’s Diversity Index highlighted the significant effect of the factor Belt (Table 3). The SNK test for the factor Belt (nested in Habitat ×Site, SE = 0.0950) highlighted at the rocky site R1 that the Belt 1 (0-50 m from the seashore) had a higher H’ than Belt 2 and Belt 3, which showed similar levels (Belt1>Belt2=Belt3), whereas at the other two rocky sites R2 and R3, Belt1<Belt2=Belt3. At the two sandy sites D1 and D2, no significant differences were highlighted in H’ amongst the three Belts (Belt1=Belt2=Belt3). Finally, at the sandy site D3, H’ showed Belt1<Belt2=Belt3.

## Discussion

The majority of contributions on soil salinity in Mediterranean coastal environments have dealt with laboratory measurements of conductivity on soil samples collected mainly on dunes (Molina et al. 2003, Carboni et al. 2011, Angiolini et al. 2013, Fenu et al. 2013, Ruocco et al. 2014,Ciccarelli et al. 2016, Cusseddu et al. 2016), whereas in this research, we present a contribution, based on in situ measurements. Here for the first time, the soil salinity gradient is measured in situ for sandy and rocky shores in north-western Sardinia, the second-largest Mediterranean island. Our data show that salinity is very low in the studied sandy soils, confirming what was already reported by Molina et al. (2003): “it is notable that, despite being the closest to the sea, the soils upon which psammophilous communities develop are those that have the lowest salinity”.

Soils of the rocky shores have a medium salinity level (following the classification proposed by the U.S. Salinity Laboratory Staff (1954) with relevant differences throughout the year but, more importantly, along the seashore-inland gradient.

Species number and biodiversity index follows an opposite trend with respect to soil salinity (with the relevant exception for H’ at rocky site R1), both being higher at the vegetation belt more distant from the seashore, where soil salinity is lower. Our results have several implications. First, this work sustains the hypothesis that the spatial soil salinity gradient is maintained throughout the year as here assessed and probably during years since no significant severe dry/wet period variations are currently verified for Sardinia (Falzoi S et al. 2019). Secondly, patterns of species numbers and biodiversity index are influenced by the distance from the seashore at both the investigated habitats and generally vary in a similar way (i.e. increasing from the seashore to inland areas), with some exceptions amongst different sites. Furthermore, whereas Belt 2 and Belt 3 show often similar levels of species richness and diversity (with the only exception of site R3 for the number of species), it is noteworthy that the Belt closer to the seashore (Belt 1) has often a significantly lower number of species (at sites R3 and D3) or diversity index (sites R2, R3 and D3) than the other two Belts: this means that this Belt is very selective for vascular plants and that, not only it hosts fewer species than the other two Belts, but also its diversity levels are lower (Torca et al. 2019), with the only exception of site R1. As a third point, this study gives the right importance to rocky coasts for the conservation of plant diversity in the Mediterranean Basin, even if their value has been often undervalued, whereas that of dunes has been greatly underlined (Feola et al. 2011), also because they are under serious threat (Prisco et al. 2013). In fact, in the last decades, coastal Mediterranean dunes have undergone a dramatic reduction and loss of diversity caused by mass tourism and severe trampling (Farris et al. 2010, Farris et al. 2013) with consequent fragmentation and decrease of plant populations (Budroni et al. 2014). Contrarily, the change of socio-economic conditions in Mediterranean regions caused a rapid land-use change in rocky areas, where traditional sylvo-pastoral systems have experienced massive encroachment (Farris et al. 2010) with loss of open vegetation and decline of species linked to semi-natural habitats (Farris et al. 2009, Pisanu et al. 2009, Pisanu et al. 2012).

## Conclusions

In conclusion, our data confirm that, also in insular Mediterranean environments, there are coastal gradients of soil salinity from the seashore to inland areas and that the distance from the coastline is the variable explaining not only the zonation of plant communities, as shown by Ruocco et al. (2014), but also species richness and diversity. Soil salinity was strongly affected by habitats, being average at the rocky coasts and negligible at the sandy shores. Site effect was not significant for both soil salinity and plant richness and diversity, whereas distance from the sea (Belt) was a major factor significantly influencing the response variables here analysed.

## Supplementary Material

B6A033C2-3AEA-5D04-8421-C4A67F091CBB 10.3897/BDJ.9.e71247.suppl1 Supplementary material 1 Gradients of salinity and plant community richness and diversity in Mediterranean coastal environments Data type Table - floristic data Brief description Frequency of 89 vascular plants in three belts (B1-B3) in three rocky sites (R1-R3) and three dune sites (D1-D3) in NW Sardinia. At each combination of Site x Belt, five transects were carried out, therefore frequency of each species is expressed as follows: 0.2 = presence in one transect; 0.4 = presence in two transects; 0.6 = presence in three transects; 0.8 = presence in four transects; 1 = presence in five transects. Plant names follow the last edition of the Italian Vascular Flora Check-List (Bartolucci et al. 2018). Biological (P = Phanerophytes, NP = Nano-phanerophytes, Ch = Chamaephytes, H = Hemicryptophytes, G = Geophytes and T = Therophytes) and chorologic forms were derived from Pignatti (Pignatti 1982). File: oo_559853.docx https://binary.pensoft.net/file/559853 Alfredo Maccioni1*, Luisa Canopoli2, Valeria Cubeddu1, Elisabetta Cucca1, Simone Dessena1, Samuele Morittu1, Rossella Filigheddu1, Bachisio Mario Padedda3, Emmanuele Farris1

## Figures and Tables

**Table 1.** Soil salinity (g/l) at the six study sites in north-western Sardinia, Italy: three rocky sites (Coscia di Donna - R1; La Frana - R2 and Cala Barca - R3) and three sandy sites (Badesi - D1; Platamona - D2 and Porto Ferro - D3). Three belts per site (B1 = 0-50 m from the sea; B2 = 51-100 m; B3 = 101-150 m) were sampled at three times in 2013 (T1 = April; T2 = August; T3 = December). ^1^Classification of soil salinity according to U.S. Salinity Laboratory Staff 1954: low= < 0.2 [salt concentration (g/l)] and < 0.25 [electrical conductivity (dS/m)]; medium= 0.2-0.5[salt concentration (g/l)] and 0.25-0.75 [electrical conductivity (dS/m)]; high=0.5-1.5 [salt concentration (g/l)] and 0.75-2.25 [electrical conductivity (dS/m)]; very high= 1.5-3.0 [salt concentration (g/l)] and 2.25-5.50 [electrical conductivity (dS/m)].

	**T1**				**T2**				**T3**		
	**B1**	**B2**	**B3**		**B1**	**B2**	**B3**		**B1**	**B2**	**B3**
**R1**	0.44±0.10	0.30±0.05	0.23±0.03		0.55±0.08	0.29±0.05	0.38±0.04		0.22±0.02	0.21±0.04	0.16±0.02
**R2**	0.40±0.07	0.23±0.02	0.28±0.05		0.29±0.04	0.23±0.06	0.27±0.04		0.26±0.03	0.19±0.04	0.15±0.02
**R3**	0.42±0.04	0.26±0.03	0.25±0.03		0.42±0.05	0.26±0.05	0.33±0.02		0.24±0.02	0.20±0.02	0.15±0.02
**D1**	0.05±0.01	0.06±0.02	0.05±0.01		0.05±0.01	0.02±0.01	0.02±0.00		0.01±0.00	0.03±0.01	0.03±0.00
**D2**	0.02±0.00	0.04±0.01	0.02±0.01		0.01±0.01	0.02±0.01	0.02±0.01		0.05±0.02	0.05±0.01	0.04±0.01
**D3**	0.03±0.00	0.05±0.01	0.04±0.00		0.03±0.01	0.02±0.00	0.02±0.01		0.03±0.01	0.04±0.01	0.03±0.01

**Table 2.** Three-way ANOVAs, testing the differences in soil salinity (g/l) between two habitats (Rocky vs. Sandy), three sites per habitat and three belts per site (Belt 1 = 0-50 m from the sea; Belt 2 = 51-100 m; Belt 3 = 101-150 m) at three sampling times in 2013 (T1 = April; T2 = August; T3 = December). N = 10 measurements were taken at each combination Time × Habitat × Site × Belt. Significant values are shown in bold.

**Source of variation**	**df**	**Time 1**				**Time 2**				**Time 3**		
		MS	F	P		MS	F	P		MS	F	P
Habitat	1	185.6975	311.55	**0.0001**		260.9545	120.86	**0.0004**		103.0304	257.81	**0.0001**
Site	4	0.5960	0.56	0.6976		2.1592	1.95	0.1668		0.3996	0.68	0.6206
Belt	12	1.0687	3.04	**0.0007**		1.1078	2.68	**0.0026**		0.5901	2.76	**0.0019**
Residual	162	0.3518				0.4139				0.2137		
Transformation		ArcSin (%)				ArcSin (%)				ArcSin (%)		
Cochran’s C-test		0.2725 (< 0.01)				0.1503 n.s.				0.1377 n.s.		

**Table 3.** Three-way ANOVAs, testing the differences in the number of species and the Shannon and Weaver's Diversity Index (H’) between two habitats (D = sandy vs. R = rocky), three sites per habitat (D1 = Li Junchi, D2 = Platamona, D3 = Porto Ferro, for sandy sites; R1 = Coscia di Donna, R2 = La Frana, R3 = Cala Barca, for rocky sites) and three belts per site (Belt 1 = 0-50 m from the sea; Belt 2 = 51-100 m; Belt 3 = 101-150 m). N = 5 point transects (100 points each) were taken at each combination Habitat × Site × Belt. Significant values are shown in bold.

**Source of variation**	**df**	**No. of species**				**Shannon and Weaver's Diversity Index (H’)**		
		MS	F	P		MS	F	P
Habitat	1	45.5111	4.74	0.0952		0.0673	0.13	0.7383
Site	4	9.6111	0.54	0.7075		0.5243	1.51	0.2602
Belt	12	17.7000	5.27	**< 0.0001**		0.3469	7.70	**< 0.0001**
Residual	72	3.3556				0.0451		
Transformation		none				none		
Cochran’s C-test		0.1821 n.s.				0.1680 n.s.		
