# Supplementary material for: Gradients of salinity and plant community richness and diversity in two different Mediterranean coastal ecosystems in NW Sardinia
Source: Biodivers Data J. 2021 Nov 19;9:e71247. doi: 10.3897/BDJ.9.e71247 (PMC8626409; doi:10.3897/BDJ.9.e71247)
Supplement: Supplementary material 1 — Gradients of salinity and plant community richness and diversity in Mediterranean coastal environments [file bdj-09-e71247-s001.docx]

**Gradients of salinity and plant community richness and diversity in Mediterranean coastal environments**

**Alfredo Maccioni^1*^, Luisa Canopoli^2^, Valeria Cubeddu^1^, Elisabetta Cucca^1^, Simone Dessena^1^, Samuele Morittu^1^, Rossella Filigheddu^1^, Bachisio Mario Padedda^3^, Emmanuele Farris^1^**

^1^ Department of Chemistry and Pharmacy, University of Sassari, Via Piandanna 4, - 07100 Sassari, Italy. alfredomaccioni87@gmail.com - orcid id: 0000-0002-9266-9523; val.cubeddu@gmail.com; e.cucca23@gmail.com; simondss@gmail.com; samuele.morittu@gmail.com; filighed@uniss.it; emfa@uniss.it - orcid id: 0000-0002-9843-5998.

^2^ School of Water, Energy and Environment, Cranfield University, Cranfield, Bedfordshire, MK43 0AL, UK. luisa.canopoli@gmail.com.

^3^ Department of Architecture, Design and Urban Planning, University of Sassari, Piazza Duomo 6, I-07041 Alghero, Italy. bmpadedda@uniss.it - orcid id: 0000-0002-0988-5613.

*Corresponding author: alfredomaccioni87@gmail.com - orcid id: 0000-0002-9266-9523;

Phone: +39 079 228675

Fax: +39 079 233600

**Supplementary Materials:** **table A.1.** Frequency of 89 vascular plants in 3 belts (B1-B3) in 3 rocky sites (R1-R3) and 3 dune sites (D1-D3) in NW Sardinia. At each combination Site x Belt 5 transects were carried out, therefore frequency of each species is expressed as follows: 0.2 = presence in 1 transect; 0.4 = presence in 2 transects; 0.6 = presence in 3 transects; 0.8 = presence in 4 transects; 1 = presence in 5 transects. Plant names follow the last edition of the Italian Vascular Flora Check-List (Bartolucci et al. 2018). Biological (P = Phanerophytes, NP = Nano-phanerophytes, Ch = Chamaephytes, H = Hemicryptophytes, G = Geophytes and T = Therophytes) and chorologic forms were derived from Pignatti (Pignatti 1982).

| **Fo. Biol.** | **Fo. Chor.** | **Name** | **R1** | | | **R2** | | | **R3** | | | **D1** | | | **D2** | | | **D3** | | |
| --- | --- | --- | --- | --- | --- | --- | --- | --- | --- | --- | --- | --- | --- | --- | --- | --- | --- | --- | --- | --- |
|  |  |  | **B1** | **B2** | **B3** | **B1** | **B2** | **B3** | **B1** | **B2** | **B3** | **B1** | **B2** | **B3** | **B1** | **B2** | **B3** | **B1** | **B2** | **B3** |
| Ch | Medit.-Atl.(Euri-) | *Achillea maritima* (L.) Ehrend. & Y.P.Guo subsp. *maritima* |  |  |  |  |  |  |  |  |  |  |  |  |  |  |  | 1 | 1 | 0.4 |
| P | avv. | *Agave americana* L. subsp. *americana* |  |  |  |  |  |  |  |  | 0.2 |  |  |  |  |  |  |  |  |  |
| H | Endem. Sar(-Cor) | *Anchusa crispa* Viv. subsp. *maritima* (Vals.) Selvi & Bigazzi |  |  |  |  |  |  |  |  |  | 0.2 |  | 0.2 |  |  |  |  |  |  |
| H | W-Medit | *Anthemis maritima* L. subsp. *maritima* |  |  |  |  |  |  |  |  |  |  |  |  |  |  |  |  | 0.2 |  |
| G | Steno-Medit. | *Arisarum vulgare* O.Targ.Tozz. subsp. *vulgare* |  |  |  | 1 | 1 | 0.8 |  |  |  |  |  |  |  |  |  |  |  |  |
| Ch | W-Europ | *Armeria pungens* (Link) Hoffmanns. & Link |  |  |  |  |  |  |  |  |  | 1 | 1 | 1 |  |  |  |  |  |  |
| G | Steno-Medit.-Occid. | *Arum pictum* L.f. subsp. *pictum* |  |  |  |  |  |  | 0.2 | 0.4 |  |  |  |  |  |  |  |  |  |  |
| G | Steno-Medit. | *Asparagus acutifolius* L. |  |  | 0.4 |  |  |  |  |  |  | 0.4 | 0.8 |  |  |  | 0.2 |  |  |  |
| G | Steno-Medit. | *Asphodelus ramosus* L. subsp. *ramosus* | 0.4 |  |  | 0.2 | 0.2 |  |  | 0.2 | 0.2 |  |  |  |  |  |  |  |  |  |
| NP | Endem. Sar (-Cor) | *Astragalus terraccianoi* Vals. |  |  |  |  |  |  |  | 0.6 | 1 |  |  |  |  |  |  |  |  |  |
| H | Endem. Ital. | *Astragalus thermensis* Vals. |  |  |  |  |  |  |  |  |  | 0.6 | 0.2 | 0.6 | 0.2 | 0.4 | 0.2 |  |  |  |
| T | Medit.-Turan. | *Brachypodium distachyon* (L.) P.Beauv. |  |  |  |  |  |  | 0.2 | 0.2 | 0.6 |  |  |  |  |  |  |  |  |  |
| T | Medit.-Atl.(Steno-) | *Cakile maritima* Scop. *maritima* |  |  |  |  |  |  |  |  |  |  | 0.4 |  |  |  |  |  |  |  |
| G | Euri-Medit. | *Calamagrostis arenaria* (L.) Roth subsp. *arundinacea* (Husn.) Banfi, Galasso & Bartolucci |  |  |  |  |  |  |  |  |  | 0.2 | 0.6 |  |  |  | 0.2 | 0.8 | 0.2 | 0.6 |
| Ch | Medit. | *Camphorosma monspeliaca* L. subsp. *monspeliaca* | 1 | 0.6 |  | 0.8 | 1 | 1 |  |  |  |  |  |  |  |  |  |  |  |  |
| H | Steno-Medit. | *Carlina corymbosa* L. | 0.2 | 0.8 | 0.6 | 0.8 | 1 | 0.8 |  |  |  |  |  |  |  |  |  |  |  |  |
| Ch | avv | *Carpobrotus acinaciformis* (L.) L. Bolus |  |  |  |  |  |  |  |  |  |  | 0.4 | 0.2 | 0.4 | 1 | 1 |  | 0.6 | 1 |
| T | Medit.-Atl.(Euri-) | *Catapodium balearicum* (Willk.) H. Scholz |  |  |  |  |  |  |  | 0.2 | 1 |  |  |  |  |  |  |  |  |  |
| Ch | Endemic | *Centaurea horrida* Badarò | 0.6 | 0.8 |  |  |  |  |  | 0.4 | 0.6 |  |  |  |  |  |  |  |  |  |
| T | Paleotemp. | *Centaurium erythraea* Rafn |  |  |  |  |  |  |  |  | 0.2 |  |  |  |  |  |  |  |  |  |
| NP | Steno-Medit.-Occid. | *Chamaerops humilis* L. |  |  |  |  |  |  |  |  | 0.2 |  |  |  |  |  |  |  |  |  |
| NP | Steno-Medit. | *Cistus creticus* L. subsp. *eriocephalus* (Viv.) Greuter & Burdet |  |  |  |  |  |  |  |  |  |  |  |  |  |  |  |  |  | 1 |
| NP | Steno-Medit. | *Cistus* *monspeliensis* L. |  |  | 0.6 |  |  |  |  |  |  |  |  |  |  |  |  |  |  | 0.2 |
| NP | Steno-Medit. | *Cistus salviifolius* L. |  | 1 | 1 |  |  |  |  |  |  |  |  |  |  |  |  |  |  | 0.2 |
| P | Euri-Medit. | *Clematis flammula* L. |  |  |  |  |  |  |  |  |  |  |  |  |  |  | 0.6 |  |  |  |
| G | Cosmop. | *Convolvulus soldanella* L. |  |  |  |  |  |  |  |  |  |  |  |  |  |  |  | 0.6 | 1 |  |
| Ch | Steno-Medit. | *Crithmum maritimum* L. |  |  |  |  |  |  |  |  |  |  |  |  |  |  |  |  |  | 0.4 |
| Ch | Steno-Medit. | *Crucianella maritima* L. |  |  |  |  |  |  |  |  |  |  | 0.4 |  |  |  |  | 0.2 | 1 | 1 |
| H | Steno-Medit. | *Dactylis glomerata* L. *hispanica* (Roth) Nyman |  |  |  |  |  |  |  |  | 0.8 |  |  |  |  |  |  |  |  |  |
| H | Paleotemp. | *Daucus carota* L. |  |  |  |  |  |  |  |  |  |  |  |  |  |  | 0.2 |  |  |  |
| H | Euri-Medit. | *Echinophora spinosa* L. |  |  |  |  |  |  |  |  |  |  |  |  | 0.2 |  |  |  |  |  |
| H | Steno-Medit. | *Echium vulgare* L. |  |  |  |  |  |  |  |  |  |  |  |  | 0.4 | 0.6 | 0.4 |  |  |  |
| NP | Steno-Medit.-Nordoccid. | *Ephedra distachya* L. |  |  |  |  |  |  |  |  |  | 0.4 | 1 | 1 |  |  | 0.6 |  |  |  |
| G | Medit.-Atl.(Euri-) | *Eryngium maritimum* L. |  |  |  |  |  |  |  |  |  | 0.2 |  |  | 1 | 0.2 |  | 1 | 0.6 | 0.4 |
| NP | Steno-Medit. | *Euphorbia characias* L. |  |  |  |  |  |  |  |  | 0.2 |  |  |  |  |  |  |  |  |  |
| T | Subcosmop. | *Euphorbia peplus* L. |  |  |  | 1 | 1 | 1 |  |  |  |  | 0.4 | 0.2 |  |  |  |  |  |  |
| Ch | W-Medit. | *Euphorbia pithyusa* L. subsp. *pithyusa* |  |  |  | 0.8 | 0.6 | 0.6 | 1 | 1 | 1 |  |  |  |  |  |  |  |  |  |
| H | Steno-Medit. | *Euphorbia terracina* L. |  |  |  |  |  |  |  |  |  |  |  |  | 1 | 0.8 | 0.4 |  |  |  |
| T | Steno-Medit. | *Fumaria bicolor* Sommier |  |  |  | 0.4 |  | 0.2 |  |  |  |  |  | 0.6 |  |  |  |  |  |  |
| NP | Endemic | *Genista corsica* (Loisel.) DC. |  | 0.2 | 0.4 |  |  |  |  |  |  |  |  |  |  |  |  |  |  |  |
| T | Eurasiat. | *Geranium robertianum* L. |  |  |  |  |  |  |  |  |  | 0.2 | 0.2 |  |  |  |  |  |  |  |
| Ch | Euri-Medit. | *Helichrysum italicum* (Roth) G.Don subsp. *tyrrhenicum* (Bacch., Brullo & Giusso) Herrando, J.M.Blanco, L.Sáez & Galbany | 0.8 | 0.4 | 1 | 1 | 1 | 1 | 1 | 1 | 1 | 0.2 |  |  |  | 0.2 | 0.2 |  | 0.2 | 1 |
| T | Paleotemp. | *Hypecoum procumbens* L. subsp. *procumbens* |  |  |  |  |  |  |  | 0.2 |  |  |  | 0.2 |  |  |  |  |  |  |
| T | Euri-Medit. | *Hypochaeris glabra* L. |  |  |  |  |  |  | 0.2 |  |  |  |  |  |  |  |  |  |  |  |
| P | Euri-Medit. | *Juniperus macrocarpa* Sm. |  |  |  |  |  |  |  |  |  |  |  |  |  | 0.2 | 1 |  |  | 0.6 |
| P | W-Medit. | *Juniperus turbinata* Guss. | 0.2 |  | 0.6 | 0.4 | 0.6 | 0.2 |  | 0.6 | 1 |  |  |  |  |  |  |  |  | 0.4 |
| T | Euri-Medit. | *Lagurus ovatus* L. subsp. *ovatus* |  |  |  |  |  |  |  |  |  |  | 0.4 | 1 |  | 0.2 | 0.8 |  |  |  |
| NP | Steno-Medit. | *Lavandula stoechas* L. subsp. *stoechas* |  |  | 0.6 |  |  |  |  |  |  |  |  |  |  |  |  |  |  |  |
| Ch | Endemic | *Limonium acutifolium* (Rchb.) Salmon subsp. *acutifolium* |  |  |  |  | 0.6 | 0.4 | 1 | 1 | 1 |  |  |  |  |  |  |  |  |  |
| Ch | Endemic | *Limonium acutifolium* (Rchb.) Salmon subsp. *nymphaeum* (Erben) Arrigoni | 0.2 |  |  |  |  |  |  |  |  |  |  |  |  |  |  |  |  |  |
| H | Steno-Medit. | *Lobularia maritima* (L.) Desv. | 0.6 | 0.4 |  | 0.4 | 0.2 | 0.6 |  |  |  |  |  | 1 |  | 0.2 | 0.8 |  |  |  |
| Ch | Steno-Medit. | *Lotus cytisoides* L. | 1 | 0.4 |  | 1 | 1 | 1 |  |  |  | 1 | 0.6 | 0.6 | 1 | 0.8 | 0.8 | 0.6 | 1 | 1 |
| H | Medit.-Atl.(Steno-) | *Matthiola sinuata* (L.) W.T.Aiton |  |  |  |  |  |  |  |  |  |  | 0.6 |  | 0.6 | 0.2 |  | 0.2 | 0.6 |  |
| T | Steno-Medit. | *Matthiola tricuspidata* (L.) R. Br. |  |  |  |  |  |  |  |  |  | 0.4 |  |  |  |  |  |  |  |  |
| Ch | Euri-Medit. | *Medicago marina* L. |  |  |  |  |  |  |  |  |  |  |  |  | 0.8 | 0.4 |  |  | 0.6 |  |
| G | Steno-Medit. | *Moraea sisyrinchium* (L.) Ker Gawl. |  |  |  |  |  |  | 0.2 |  | 0.6 |  |  |  |  |  |  |  |  |  |
| P | Steno-Medit. | *Olea europae*a L. |  | 0.4 | 0.8 |  |  |  |  |  |  |  |  |  |  |  |  |  |  |  |
| Ch | Euri-Medit.-Occid. | *Ononis natrix* L. subsp. *natrix* |  |  |  |  |  |  |  |  |  |  |  |  |  |  |  |  |  | 0.2 |
| G | Steno-Medit. | *Pancratium maritimum* L. |  |  |  |  |  |  |  |  |  | 0.8 | 0.4 |  | 1 | 0.4 |  | 1 | 1 | 0.4 |
| T | Medit.-Turan. | *Papaver dubium* L. |  |  |  |  |  |  |  |  |  |  |  | 0.6 |  |  |  |  |  |  |
| P | Steno-Medit. | *Pinus halepensis* Mill. |  |  |  |  |  |  |  |  |  |  |  |  |  |  |  |  |  | 0.2 |
| P | Steno-Medit. | *Pistacia lentiscus* L. | 0.8 | 0.6 | 1 |  |  |  |  | 0.4 | 0.6 |  |  |  |  |  |  |  |  | 0.2 |
| H | Subcosmop. | *Polygonum maritimum* L. |  |  |  |  |  |  |  |  |  |  |  |  |  |  |  | 0.2 |  |  |
| H | Steno-Medit. | *Reichardia picroides* (L.) Roth |  | 0.4 |  |  |  | 0.2 |  |  |  |  |  |  |  |  |  |  |  |  |
| T | Steno-Medit.-Occid. | *Rostraria pubescens* (Lam.) Trin. |  |  |  |  |  |  |  |  |  |  |  |  |  |  | 0.6 |  |  |  |
| P | Steno-Medit. | *Rubia peregrina* L. |  |  |  | 0.2 |  |  |  |  |  | 0.4 |  |  |  | 0.2 |  |  |  |  |
| T | Paleotemp. | *Salsola kali* L. |  |  |  |  |  |  |  |  |  |  |  |  |  |  | 0.2 |  |  |  |
| NP | Steno-Medit. | *Salvia rosmarinus* Spenn. | 0.2 |  |  |  |  |  |  |  |  |  |  |  |  |  |  |  |  |  |
| H | Steno-Medit. | *Salvia verbenaca* L. |  |  |  | 0.2 |  |  |  |  |  |  |  |  |  |  |  |  |  |  |
| G | Euri-Medit. | *Scirpoides holoschoenus* (L.) Soják |  |  |  |  |  |  |  |  |  |  |  |  |  | 0.6 |  |  |  |  |
| Ch | Steno-Medit. | *Sedum dasyphyllum* L. |  |  |  |  |  |  |  |  |  |  |  | 0.2 |  |  |  |  |  |  |
| T | Steno-Medit. | *Senecio leucanthemifolius* Poir. subsp. *leucanthemifolius* | 1 | 0.2 |  |  | 1 | 0.4 |  |  | 0.2 | 0.8 | 0.2 | 0.6 | 0.2 |  |  |  |  |  |
| T | Steno-Medit. | *Silene niceensis* All. |  |  |  |  |  | 0.2 |  |  |  |  |  |  | 0.2 | 0.6 | 0.2 |  |  |  |
| T | Endemic | *Silene nummica* Vals. |  |  |  |  |  |  |  |  |  | 0.4 | 0.2 | 1 |  |  |  |  |  |  |
| H | Endemic | *Silene succulenta* Forssk. subsp. *corsica* (DC.) Nyman |  |  |  |  |  |  |  |  |  |  |  |  | 0.2 |  |  |  |  |  |
| H | Steno-Medit. | *Sixalix atropurpurea* (L.) Greuter & Burdet |  |  |  |  |  |  |  |  |  |  |  |  |  |  | 0.2 |  |  |  |
| G | Steno-Medit. | *Sonchus bulbosus* (L.) N. Kilian & Greuter subsp. *bulbosus* | 0.4 | 1 | 1 |  |  |  |  |  |  |  | 0.6 |  |  |  | 0.8 | 0.2 | 0.8 |  |
| G | Subtrop. | *Sporobolus virginicus* (L.) Kunth |  |  |  |  |  | 0.2 |  |  |  | 0.8 | 0.8 |  | 0.2 |  |  | 0.2 | 1 | 1 |
| Ch | Steno-Medit. | *Stachys major* (L.) Bartolucci & Peruzzi |  |  | 0.4 |  |  |  |  |  |  |  | 0.2 |  |  |  |  |  |  |  |
| T | Cosmop. | *Stellaria media* (L.) Vill. |  |  |  |  |  |  |  |  |  |  |  | 0.4 |  |  |  |  |  |  |
| H | Cosmop. | *Taraxacum* sect. *Taraxacum* |  |  |  |  |  |  |  | 0.2 |  |  |  |  |  |  |  |  |  |  |
| G | Euri-Medit. | *Thinopyrum junceum* (L.) Á.Löve |  |  |  |  |  |  |  |  |  |  |  |  | 1 | 0.6 |  | 0.4 | 1 | 0.2 |
| Ch | S-Medit. | *Thymelaea hirsuta* (L.) Endl. |  |  | 0.4 |  |  |  |  |  |  |  |  |  |  |  |  |  |  |  |
| Ch | Steno-Medit. | *Thymelaea tartonraira* (L.) All. subsp. *tartonraira* |  |  |  |  |  |  |  |  |  |  |  |  |  |  |  |  |  | 1 |
| T | Paleotemp. | *Trifolium arvense* L. |  |  |  |  |  |  |  | 0.2 |  |  |  |  |  |  |  |  |  |  |
| G | Steno-Medit. | *Umbilicus horizontalis* (Guss.) DC. |  |  |  |  |  |  |  |  |  |  | 0.2 | 0.2 |  |  | 0.2 |  |  |  |
| T | Steno-Medit. | *Valantia muralis* L. |  |  |  |  |  |  |  |  | 0.2 |  |  |  |  |  |  |  |  |  |
| T | Steno-Medit. | *Valerianella macrocarpa* Loisel. |  |  |  |  |  |  |  |  |  |  |  | 0.4 |  |  |  |  |  |  |
| T | - | Not determined |  |  |  |  |  |  |  |  |  | 0.2 |  |  |  |  |  |  |  |  |
| **Total number of species per site** | | | **22** | | | **18** | | | **23** | | | **33** | | | **30** | | | **27** | | |

**References**

Bartolucci F, Peruzzi L, Galasso G, Albano A, Alessandrini A, Ardenghi NMG, Astuti G, Bacchetta G, Ballelli S, Banfi E, Barberis G, Bernardo L, Bouvet D, Bovio M, Cecchi L, Di Pietro R, Domina G, Fascetti S, Fenu G, Festi F, Foggi B, Gallo L, Gottschlich G, Gubellini L, Iamonico D, Iberite M, Jiménez-Mejías P, Lattanzi E, Marchetti D, Martinetto E, Masin RR, Medagli P, Passalacqua NG, Peccenini S, Pennesi R, Pierini B, Poldini L, Prosser F, Raimondo FM, Roma-Marzio F, Rosati L, Santangelo A, Scoppola A, Scortegagna S, Selvaggi A, Selvi F, Soldano A, Stinca A, Wagensommer RP, Wilhalm T, Conti F (2018) An updated checklist of the vascular flora native to Italy. Plant Biosyst 152: 179-303. doi:10.1080/11263504.2017.1419996

Pignatti S (1982) Flora d’Italia. 3 vols. Edagricole, Italy
